# Tract Specificity of Age Effects on Diffusion Tensor Imaging Measures of White Matter Health

**DOI:** 10.3389/fnagi.2021.628865

**Published:** 2021-03-15

**Authors:** Stephanie Matijevic, Lee Ryan

**Affiliations:** Cognition and Neuroimaging Laboratory, Department of Psychology, University of Arizona, Tucson, AZ, United States

**Keywords:** aging, diffusion tensor imaging, white matter, MRI, sex

## Abstract

Well-established literature indicates that older adults have poorer cerebral white matter integrity, as measured through diffusion tensor imaging (DTI). Age differences in DTI have been observed widely across white matter, although some tracts appear more sensitive to the effects of aging than others. Factors like APOE ε4 status and sex may contribute to individual differences in white matter integrity that also selectively impact certain tracts, and could influence DTI changes in aging. The present study explored the degree to which age, APOE ε4, and sex exerted global vs. tract specific effects on DTI metrics in cognitively healthy late middle-aged to older adults. Data from 49 older adults (ages 54–92) at two time-points separated by approximately 2.7 years were collected. DTI metrics, including fractional anisotropy (FA) and mean diffusivity (MD), were extracted from nine white matter tracts and global white matter. Results showed that across timepoints, FA and MD increased globally, with no tract-specific changes observed. Baseline age had a global influence on both measures, with increasing age associated with lower FA and higher MD. After controlling for global white matter FA, age additionally predicted FA for the genu, callosum body, inferior fronto-occipital fasciculus (IFOF), and both anterior and posterior cingulum. Females exhibited lower global FA on average compared to males. In contrast, MD was selectively elevated in the anterior cingulum and superior longitudinal fasciculus (SLF), for females compared to males. APOE ε4 status was not predictive of either measure. In summary, these results indicate that age and sex are associated with both global and tract-specific alterations to DTI metrics among a healthy older adult cohort. Older women have poorer white matter integrity compared to older men, perhaps related to menopause-induced metabolic changes. While age-related alterations to white matter integrity are global, there is substantial variation in the degree to which tracts are impacted, possibly as a consequence of tract anatomical variability. The present study highlights the importance of accounting for global sources of variation in DTI metrics when attempting to investigate individual differences (due to age, sex, or other factors) in specific white matter tracts.

## Introduction

Cerebral white matter undergoes multiple changes throughout healthy aging. Dysmyelination, axonal degeneration, ischemia, and inflammatory processes associated with aging may compromise the structural integrity of axonal fibers within cerebral white matter bundles (Peters, 2009). The overall impact of these various pathological processes on white matter microstructure can be indirectly assessed through the use of diffusion tensor imaging (DTI; Alexander et al., 2007). Based on the principle that intact myelinated fibers restrict the movement of water within and especially across fiber bundles, DTI derives estimates of diffusion magnitude and directionality that can serve as proxies for white matter microstructural integrity. The DTI-derived metric mean diffusivity (MD) expresses the average amount of diffusion occurring within an image voxel. Fractional anisotropy (FA) is a ratio reflecting the directional consistency of diffusion, with higher values indicating that diffusion within a voxel is primarily restricted to one direction or, in cases like crossing fibers, to two directions (O’Donnell and Westin, 2011). When calculated for voxels containing white matter tissue, both MD and FA are influenced by attributes of individual axons and fiber bundles or tracts, such as the density of myelin and tract compactness, as well as the size and composition of extracellular components (Alexander et al., 2007). Opposing correlations are typically seen between these measures and age in adulthood. Increasing age is typically associated with higher MD and lower FA (Lebel et al., 2012; Cox et al., 2016; Storsve et al., 2016; for review, see Madden et al., 2009), likely reflecting the impact of a combination of age-related phenomena including dysmyelination, glial-induced inflammatory activity, dysfunctional glial clearance of extracellular debris, axonal degeneration, and ischemic injury (Peters, 2009; Raj et al., 2017; Chapman and Hill, 2020).

Axial diffusivity (AD) and radial diffusivity (RD) are additional DTI metrics that may correlate more specifically to certain types of white matter pathology. AD represents diffusion occurring along the length of white matter fibers, while RD is calculated as the amount of diffusion perpendicular to the main directional axis of fibers. Early studies of optic nerve fiber damage (Song et al., 2003, 2005) indicated that increases in AD and RD values correlate with axonal degradation and dysmyelination, respectively. However, a more diverse set of animal models for multiple sclerosis (Budde et al., 2007, 2009; Falangola et al., 2014) and spinal cord injury (Budde et al., 2007) have revealed that AD is less specific for axonal degradation and RD is less specific for dysmyelination when multiple pathologies co-occur (for review, see Winklewski et al., 2018). Human studies have consistently noted increases in RD associated with increasing age in late adulthood (Bennett et al., 2010; Brickman et al., 2012; Marstaller et al., 2015), while the relationship between age and AD is less straightforward, as both increases and decreases in AD have been demonstrated among older adults (Bennett et al., 2010; Burzynska et al., 2010).

A long-standing question regarding cerebral white matter abnormalities in aging is the degree to which these changes can be characterized as uniform across the brain vs. regionally specific. Twin studies have revealed that genetics account for over half of the global variation in DTI metrics within middle-aged to older adults (Vuoksimaa et al., 2017; Gustavson et al., 2019), as well as additional tract-specific variation in late adulthood (Kanchibhotla et al., 2014; Vuoksimaa et al., 2017; Hatton et al., 2018; Gustavson et al., 2019). Gustavson et al. (2019) noted that these findings emphasize the importance of accounting for global sources of variation in white matter DTI values when attempting to examine associations between individual tracts and variables like age. Multivariate analyses of white matter tract DTI values have identified a general factor of white matter integrity capable of explaining a substantial amount of variance shared across tracts, among older adults (Penke et al., 2010; Lövdén et al., 2013; Cox et al., 2016). Cox et al. (2016) demonstrated an association between age and a general factor for FA, derived through the latent-variable analysis of tract FA values. However, this general factor did not fully account for tract-specific age associations, indicating regional variation in the magnitude of the association between age and FA. Such regional variation has been observed within the corpus callosum, as the anterior segment of the corpus callosum tends to be more sensitive to age-related FA reductions compared to posterior segments (Bendlin et al., 2010; Michielse et al., 2010; Bennett et al., 2010; Cox et al., 2016). Overall, the corpus callosum tends to show smaller alterations to both FA and MD compared to long-range association tracts (such as the superior longitudinal, inferior longitudinal, inferior fronto-occipital, and uncinate fasciculi) and limbic tracts (fornix and cingulum), but larger alterations compared to sensorimotor projection tracts such as the internal capsule (de Groot et al., 2015; Hoagey et al., 2019). However, it is important to note that tract-specific changes in white matter are inconsistent across studies. For instance, while an anterior-to-posterior gradient of age-related FA reduction has been consistently noted within the corpus callosum, there is mixed evidence as to whether this gradient applies to other regions of cerebral white matter (Brickman et al., 2012; Hoagey et al., 2019).

Genetics, lifestyle, and health factors may contribute to individual differences in white matter integrity, particularly in later life (Bender and Raz, 2015). Apolipoprotein E (APOE) ε4 allele, the strongest known genetic risk factor for the development of sporadic Alzheimer’s disease (Kline, 2012), has been shown to impact DTI metrics in older adults. Cross-sectional studies have noted that cognitively healthy older adults carrying one or more copies of the ε4 allele show greater DTI alterations compared to non-carriers, particularly in FA (for review, see Kanchibhotla et al., 2013). Among older adults, ε4 is associated with reduced FA in specific tracts including the cingulum (Smith et al., 2010; Lyall et al., 2014; Cavedo et al., 2017), the corpus callosum (Persson et al., 2006; Smith et al., 2010; Laukka et al., 2015; Cavedo et al., 2017), and the superior and inferior longitudinal fasciculi (Smith et al., 2010; Lyall et al., 2014; Cavedo et al., 2017). However, multiple other studies have reported the absence of an ε4 effect for any white matter tract (Bendlin et al., 2010; Westlye et al., 2012; Ly et al., 2014; Nyberg and Salami, 2014). One explanation for these contradictory findings is that ε4 might influence the trajectory of age-related changes in DTI metrics, rather than engendering an overall difference between ε4 carriers and non-carriers. Supporting this idea, a cross-sectional study by Ryan et al. (2011) reported an interaction between ε4 status and age in a cohort of older adults, ages 52–92. They found that ε4 carriers exhibited steeper age-related increases in the average diffusion coefficient across all white matter regions, and steeper FA decreases within frontal white matter, temporal white matter, and the genu of the corpus callosum compared to non-carriers. Similar findings of an ε4 by age interaction have been noted in other studies (Adluru et al., 2014; Williams et al., 2019), although some studies have not detected such an interaction (Nyberg and Salami, 2014; Rieckmann et al., 2016).

While APOE ε4 has been the focus of multiple studies, other potentially important factors, such as sex, have garnered relatively little attention. Middle-aged females undergo sweeping endocrine changes during menopause that likely contribute to increased prevalence of autoimmune diseases (Desai and Brinton, 2019) and potentially greater susceptibility to age-related white matter damage through enhanced dysmyelination (Klosinski et al., 2015). Indeed, a poor metabolic profile in combination with the APOE ε4 genotype has been associated with reduced cognitive performance in otherwise healthy postmenopausal women (Karim et al., 2019). Mosconi et al. (2017) examined white matter volumes for pre-, peri- and post-menopausal middle-aged women and found a significant decline in white matter volume from pre- to post-menopausal stages, providing support for the idea that menopause contributes to white matter degeneration in older women. Sex differences in white matter may also be present from birth, however, as suggested by research demonstrating an influence of sex hormones on white matter development in adolescence (Herting et al., 2012; Menzies et al., 2015; Peper et al., 2015; Ho et al., 2020). Sexual dimorphism in DTI metrics has not been consistently observed in adults, but studies that have found sex differences note that females tend to have lower FA compared to males (Lebel et al., 2010; Inano et al., 2011; van Hemmen et al., 2017; Ritchie et al., 2018), although others have reported the opposite pattern as well (O’Dwyer et al., 2012). In part, this inconsistency could stem from regional variability in the impact of sex on DTI metrics. For example, Inano et al. (2011) reported age-related changes in global measures of FA, AD, and RD, but found sex differences that were tract-specific. Significantly lower FA was observed in females compared with males in multiple regions including the splenium of the corpus callosum, the posterior internal capsule, bilateral cingulum, and bilateral superior longitudinal fasciculus (but note that FA was higher for females in the columns of the fornix). The possibility that menopause may enhance susceptibility to white matter damage, particularly in a tract-specific manner, highlights the importance of considering sex differences in DTI studies of aging.

The present study sought to characterize the degree to which age, ε4 status, and sex influence white matter integrity among older adults, and whether these differences are best characterized as global or tract-specific. DTI metrics were obtained from whole-brain white matter maps as well as tracts commonly implicated in studies of aging, including the genu, body, and splenium of the corpus callosum, the anterior and posterior cingulum bundles, the superior longitudinal fasciculi, the inferior fronto-occipital fasciculi, the fornix, and the uncinate fasciculi. Additionally, we obtained these DTI measures from our cohort at two time-points separated 2.7 years apart on average. Cross-sectional studies are limited in their ability to precisely characterize the impact of aging on white matter integrity. While longitudinal studies have generally demonstrated age-related changes to FA and MD that are similar to cross-sectional findings (Teipel et al., 2010; Sexton et al., 2014; Bender and Raz, 2015; Bender et al., 2016; Williams et al., 2019), they have also identified white matter changes that are not detected in cross-sectional studies. For example, Bender and Raz (2015) found increases in FA across a two to three year period in the body of the corpus callosum, in contrast to cross-sectional FA decreases with age in this tract at baseline. Examining how DTI metrics shift over time may provide a more precise assessment of global and tract-specific effects of risk factors on white matter integrity.

## Materials and Methods

### Participants

Forty-nine cognitively healthy older adults (ages 54–92) who participated in Ryan et al. (2011) were recruited for a second wave of data collection approximately 2.7 years after their first visit (see Table 1 for demographics). All participants were community-dwelling residents of the Tucson, Arizona region. Eligible participants were screened for neurological disorders, drug/alcohol abuse, psychiatric disorders, stroke, traumatic brain injury, and MRI complications, and scored within normal limits on a neuropsychological test battery assessing memory, executive functions, and processing speed. Informed consent was obtained following the guidelines set by the University of Arizona’s Institutional Review Board.

**Table 1:** Sample demographics.

Demographic variable	
*N*	49
Years of age at T1, mean ± SD (range)	70.4 ± 8.2 (54.1–91.8)
Months between scans, mean ± SD	32.9 ± 6.63
Sex, *N* (%)	
Male	13 (27%)
Female	36 (73%)
Years of education, mean ± SD	16.0 ± 2.6
ε4 Status, *N* (%)	
ε4 Carrier	20 (41%)
ε4 Non-carrier	29 (59%)

### APOE Genotyping

Saliva samples were obtained *via* the Oragene-DNA device (OG-500, DNA Genotek Inc., Toronto, ON, Canada) from participants and sent to the Translational Genomics Research Institute (Phoenix, AZ, USA) for genotyping. DNA was extracted from the saliva samples using the manufacturer’s protocol and genotyped for two single nucleotide polymorphisms within APOE, rs429358, and rs7412, *via* TaqMan chemistry (Thermo Fisher Scientific Inc., Waltham, MA, USA). Participants with the ε2/ε4, ε3/ε4, or ε4/ε4 genotype were categorized as ε4 carriers (*n* = 20) for this study, while ε2/ε3 and ε3/ε3 individuals were considered non-carriers (*n* = 29). No ε2 homozygotes were present in this sample.

### MR Image Acquisition

At both visits, images were obtained with a General Electric 3.0 T Signa (Milwaukee, WI, USA) MR scanner. An echo-planar DTI sequence was used to collect two non-diffusion weighted images and diffusion-weighted images in 25 directions (b-value = 1,000 s/mm^2^, 58 contiguous axial sections, 2.6 mm thickness, TE = 71 ms, TR = 13,000 ms, matrix = 96 × 96, FOV = 250 × 250 mm^2^, voxel size = 2.6 mm^3^). High resolution T1-weighted SPGR images were obtained with the following parameters: 0.7 mm thickness, TE = 2 ms, TR = 5.1 ms, flip angle = 15°, matrix = 256 × 256, FOV = 260 × 260 mm^2^. Participants with incidental MR findings, such as evidence of past stroke damage, were excluded from the study.

### Diffusion Tensor Imaging

Diffusion-weighted images were visually inspected for artifacts. Skull stripping, motion and eddy current corrections, and tensor fitting were performed with FSL tools^1^ (Smith et al., 2004). Registration of images across the two time-points followed an optimized longitudinal DTI analysis protocol detailed in Keihaninejad et al. (2013), which was developed to track white matter changes occurring in neurodegenerative conditions such as Alzheimer’s disease. Keihaninejad et al.’s (2013) protocol attempts to mitigate the sensitivity of ROI-based analyses to registration and partial volume errors, which are particularly an issue when atrophy is present, by incorporating diffusion tensor information into the registrations such that the alignment process takes into account local fiber orientation. Using DTI-TK’s^2^ tensor-based registration method (Zhang et al., 2006), a within-subject template was created for each participant by registering and averaging the tensor images from both time points. Within-subject templates from all participants were averaged together to create a group template, and each participant’s two tensor images were then normalized to this group template.

**Figure 1:**
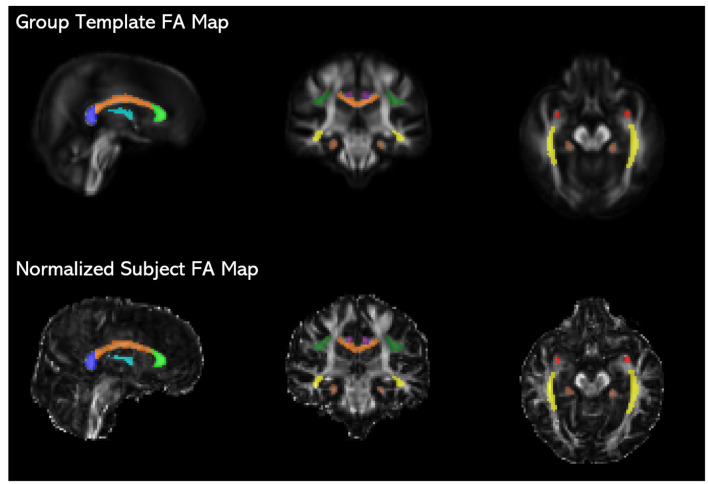
JHU ICBM-DTI-81 white matter atlas tract labels (Mori et al., 2008) registered to the study-specific group template are overlaid on fractional anisotropy (FA) maps of the group template and an individual subject’s time 1 image normalized to the group template space, showing the placement of the fornix (light blue), genu (light green), corpus callosum body (orange), splenium (blue), uncinate fasciculus (red), inferior fronto-occipital fasciculus (IFOF; yellow), anterior cingulum (purple), posterior cingulum (brown), and superior longitudinal fasciculus (SLF; dark green).

For each participant, FA, MD, RD AD maps were derived from the normalized tensor images. To extract tract-specific DTI metrics, the Johns Hopkins University ICBM-DTI-81 2 mm white matter atlas (JHU atlas; Mori et al., 2008) was registered to the study-specific group template. The JHU atlas tract labels were then used to identify regions of interest (ROIs) on the normalized DTI maps for each participant. The ROIs were further masked with a binary white matter mask created by thresholding the FA maps at a value of 0.2 to ensure that only fully-volumed white matter was included in the ROIs. ROIs (see Figure 1) included the anterior cingulum, posterior cingulum, superior longitudinal fasciculus (SLF), inferior fronto-occipital fasciculus (IFOF), fornix body, uncinate fasciculus, and the corpus callosum. The corpus callosum was segmented into the genu, callosal body, and splenium, defined in the JHU atlas as the first, second, and last thirds of the corpus callosum, respectively. The IFOF ROI comprised of the temporal and occipital portions of the tract and was inclusive of the inferior longitudinal fasciculus. FA, MD, RD, and AD values were averaged across all voxels within an ROI. For bilateral ROIs (anterior and posterior cingulum, SLF, uncinate fasciculus, and IFOF), values were extracted separately, then averaged across the left and right ROIs, since we did not have *a priori* hypotheses regarding laterality effects. Additionally, we found that the values from left and right hemispheres for each tract at baseline were correlated at *r* = 0.35 or higher, all *p*’s < 0.05. To calculate global diffusion measures, the white matter mask was applied to each DTI map—FA, MD, RD, and AD—and the values for all voxels within the mask were then averaged.

### Statistical Analyses

To determine whether the impact of the independent variables on DTI metrics are best characterized as global or tract-specific, we compared the outcomes from two sets of mixed factor MANOVAs, with and without the inclusion of global DTI measures as covariates. This allowed us to calculate the difference in variance explained within specific tracts that was accounted for by our predictors before and after controlling for global DTI measures. Employing MANOVAs also allowed us to control for experiment-wise error due to the large numbers of tracts included in the study.

First, separate MANOVAs were conducted to predict DTI values within specific tracts, without controlling for global DTI measures. Diffusion values at time 1 and time 2 for each tract were entered as dependent variables, with time (time 1, time 2) modeled as a within-subject predictor. Mean-centered age at time point 1, sex (female, male), and APOE ε4 status (carrier, non-carrier) were included as between-subject predictors. All main effects and the interactions between time and the between-subject predictors were included in the model. Pillai’s Trace served as the test statistic in the MANOVA models, as it is more robust to inhomogeneity of covariance and variance. The second set of MANOVA models were identical to the first set but controlled for global sources of variation in DTI metrics, through the inclusion of the global DTI measures (at time 1) to their respective models as a covariate. The two sets of models were compared to identify associations with tract-specific DTI values that were independent of global white matter influences.

As a follow-up analysis for each effect identified as tract-specific, stepwise regressions were conducted for each tract. Global FA or MD was entered as a predictor into each regression model first, followed by the predictor of interest. To further explore associations between our predictors and global DTI measures, we conducted mixed factor ANOVAs. These models included the same set of predictors as the tract-specific MANOVAs—the age at time 1 (mean-centered), sex, APOE ε4 status, time, and the interactions between time and each between-subject variable. In the two separate ANOVAs, global FA and global MD at time 1 and time 2 were entered as dependent variables.

## Results

Correlations amongst global MD, RD, and AD values at time point 1 yielded Pearson *r* coefficients greater than 0.9, while correlations between these measures and global FA were smaller with coefficients ranging between 0.4 and 0.7 (see Table 2). The strong correlations between AD, RD, and MD values suggested that these measures were redundant. Thus, only MD and FA values were included in our analyses.

**Table 2:** Correlation matrix showing Pearson *r* values for associations between global diffusion tensor imaging (DTI) measures at time point 1.

	Global FA	Global RD	Global AD
Global RD	−0.685**		
Global AD	−0.432**	0.932**	
Global MD	−0.595**	0.989**	0.975**

**p < 0.01.

### Determining Tract-Specific Predictors of DTI Metrics

To determine whether the impact of the independent variables on DTI metrics are best characterized as global or tract-specific, we compared the outcomes from two sets of mixed factor MANOVAs, with and without the inclusion of global DTI measures as covariates. This allowed us to calculate the difference in variance explained within specific tracts that was accounted for by our predictors before and after controlling for global DTI measures. Employing MANOVAs also allowed us to control for experiment-wise error due to the large numbers of tracts included in the study.

First, separate MANOVAs were conducted to predict FA and MD values within specific tracts, without controlling for global DTI measures. Diffusion values at time 1 and time 2 for each tract were entered as dependent variables, with time (time 1, time 2) modeled as a within-subject predictor. Mean-centered age at time point 1, sex (female, male), and APOE ε4 status (carrier, non-carrier) were included as between-subject predictors. All main effects and the interactions between time and the between-subject predictors were included in the model. Pillai’s Trace served as the test statistic in the MANOVA models, as it is more robust to inhomogeneity of covariance and variance. The second set of MANOVA models were identical to the first set but controlled for global sources of variation in DTI metrics. Global FA and MD values at time 1 were added to the models as covariates for FA and MD values, respectively. The two sets of models were compared to identify associations with tract-specific DTI values that were independent of global white matter influences. Results from the four mixed factor MANOVAs as well as appropriate follow-up analyses for specific tracts are described below.

#### Fractional Anisotropy

Results of the MANOVAs predicting FA are found in Table 3. The tract-specific MANOVA without controlling for global FA indicated significant main effects of time, sex, and age. After controlling for global FA at time 1, the main effects of time and sex were no longer significant. The effect of age, however, remained significant. FA values were not significantly influenced by APOE ε4 status in either model, and the interactions between time and other predictor variables did not approach significance in either model.

**Table 3:** Results of the mixed MANOVAs for tract fractional anisotropy (FA) values.

	Tract FA model			Tract FA model (controlling for Global FA)		
Predictors	*F*	*p*	*Pillai’s trace*	*F*	*p*	*Pillai’s trace*
**Within-subject**		
Time	2.73	0.015	0.40	1.25	n.s.	0.24
Time * Age	2.11	n.s.	0.34	1.63	n.s.	0.29
Time * Sex	1.39	n.s.	0.25	1.41	n.s.	0.26
Time * APOE ε4	0.43	n.s.	0.10	0.42	n.s.	0.10
Time * Global FA				1.27	n.s.	0.24
**Between-subject**		
Age	6.34	<0.001	0.61	4.43	0.001	0.53
Sex	3.47	0.003	0.46	1.67	n.s.	0.29
APOE ε4	1.36	n.s.	0.25	1.34	n.s.	0.25
Global FA				9.61	<0.001	0.71

*On the left, results for the initial version of the model without controlling for global FA are shown. On the right, results for the model controlling for global FA (at time 1) are shown*.

#### Mean Diffusivity

Results of the MANOVAs predicting MD are found in Table 4. The tract-specific MANOVA without controlling for global MD showed a similar pattern to FA, indicating the main effects of time, sex, and age. After controlling for global MD, the effect of time was no longer significant. Unlike the results for FA, the main effect of age also became non-significant, while the main effect of sex remained significant. In both models, and consistent with the results for FA, APOE ε4 status did not predict MD values, and the interactions between time and other predictor variables did not approach significance.

**Table 4:** Results of the mixed MANOVAs for tract mean diffusivity (MD) values.

	Tract MD model			Tract MD model (controlling for Global MD)		
Predictors	*F*	*p*	*Pillai’s trace*	*F*	*p*	*Pillai’s trace*
**Within-subject**
Time	2.77	0.014	0.40	1.07	n.s.	0.21
Time * Age	1.51	n.s.	0.27	1.56	n.s.	0.28
Time * Sex	1.45	n.s.	0.26	1.53	n.s.	0.28
Time * APOE ε4	0.73	n.s.	0.15	0.73	n.s.	0.15
Time * Global MD				1.08	n.s.	0.21
**Between-subject**
Age	6.10	<0.001	0.60	1.43	n.s.	0.26
Sex	7.62	<0.001	0.65	6.63	<0.001	0.62
APOE ε4	1.85	n.s.	0.31	1.91	n.s.	0.32
Global MD				11.46	<0.001	0.74

*On the left, results for the initial version of the model without controlling for global MD are shown. On the right, results for the model controlling for global MD (at time 1) are shown*.

### Tract-Specific Associations Between Age and FA

**Figure 2:**
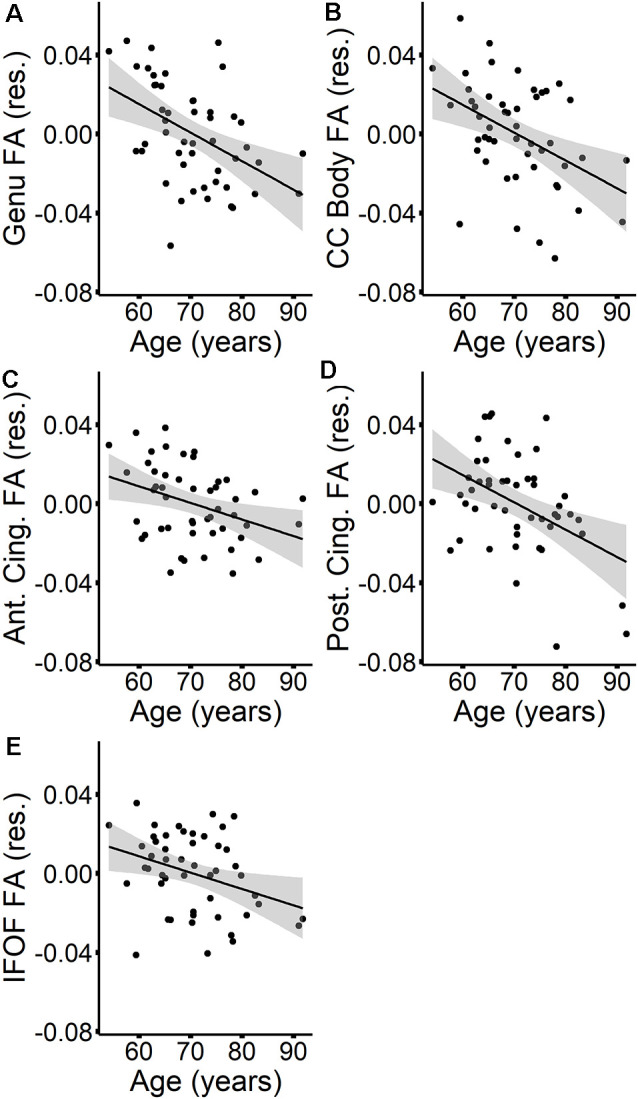
The relationships between age (at time 1) and tract FA values (at time 1)—residualized for global FA values (at time 1)—are presented in scatterplots for the genu **(A)**, callosum body **(B)**, anterior cingulum **(C)**, posterior cingulum **(D)**, and IFOF **(E)**. Confidence intervals are shown in gray.

The MANOVAs indicated that age did not predict MD in specific tracts once global MD was accounted for. However, age predicted FA in specific tracts, even after controlling for global FA. To further explore the effect of age on tract-specific FA values, we first performed Pearson correlations between tract FA values and age at time 1, which showed that increasing age was associated with lower FA in all tracts except for the SLF (see Table 5). Stepwise regressions were then performed for each tract in which global FA at time 1 was entered first as a predictor, followed by age at time 1. Results listed in Table 6, indicated a negative influence of age on FA values in the genu, callosum body, anterior cingulum, posterior cingulum, and the IFOF that went beyond individual baseline differences in global FA. The additional variance explained by age beyond global FA ranged from 6% in the IFOF to 20% in the posterior cingulum. Figure 2 depicts the relationships, for these five tracts, between age and tract FA values residualized for global FA values at time 1.

**Table 5:** Pearson correlation *r* values for associations between age and tract FA values at time 1.

Genu	Callosum body	Splenium	Fornix	Ant. cingulum	Post. cingulum	SLF	IFOF	Uncinate
−0.57**	−0.55**	−0.40**	−0.37*	−0.50**	−0.54**	−0.22	−0.48**	−0.31*

**p < 0.01; *p < 0.05.

**Table 6:** Results for stepwise regressions performed on tract FA values (at time 1).

Tract	Predictors	*β*	*t*	*p*	*F*_(2,46)_	*Adj. R*^2^	*R*^2^ change
Genu	Global FA	0.55	5.51	<0.001			
	**Age**	−**0.38**	−**3.85**	<**0.001**	**33.90**	**0.58**	**0.13**
Callosum body	Global FA	0.39	3.30	0.002			
	**Age**	−**0.42**	−**3.57**	<**0.001**	**18.03**	**0.42**	**0.16**
Splenium	Global FA	0.55	4.60	<0.001			
	Age	−0.21	−1.74	n.s.	16.85	0.40	0.04
Fornix	Global FA	0.39	2.87	0.006			
	Age	−0.24	−1.75	n.s.	8.35	0.23	0.05
Anterior cingulum	Global FA	0.63	6.37	<0.001			
	**Age**	−**0.28**	−**2.81**	**0.007**	**34.51**	**0.58**	**0.07**
Posterior cingulum	Global FA	0.18	1.42	n.s.			
	**Age**	−**0.47**	−**3.63**	**0.001**	**10.64**	**0.29**	**0.20**
SLF	Global FA	0.87	10.38	<0.001			
	Age	0.08	0.91	n.s.	57.92	0.70	0.01
IFOF	Global FA	0.62	5.96	<0.001			
	**Age**	−**0.27**	−**2.61**	**0.012**	**30.14**	**0.55**	**0.06**
Uncinate	Global FA	0.52	4.00	<0.001			
	Age	−0.13	−0.99	n.s.	11.17	0.30	0.01

*Global FA was entered into each model first, followed by age. Only results for the complete model (Global FA + Age) are included here. Standardized β coefficients, *t*-statistics, and *p*-values are shown for Global FA and Age predictors. The model-level *F* statistic and Adj. R^2^ are also reported. R^2^ change represents the amount of variance additionally explained in the model after adding Age as a predictor. Statistically significant age effects are bolded*.

### Tract-Specific Sex Differences in MD Values

**Figure 3:**
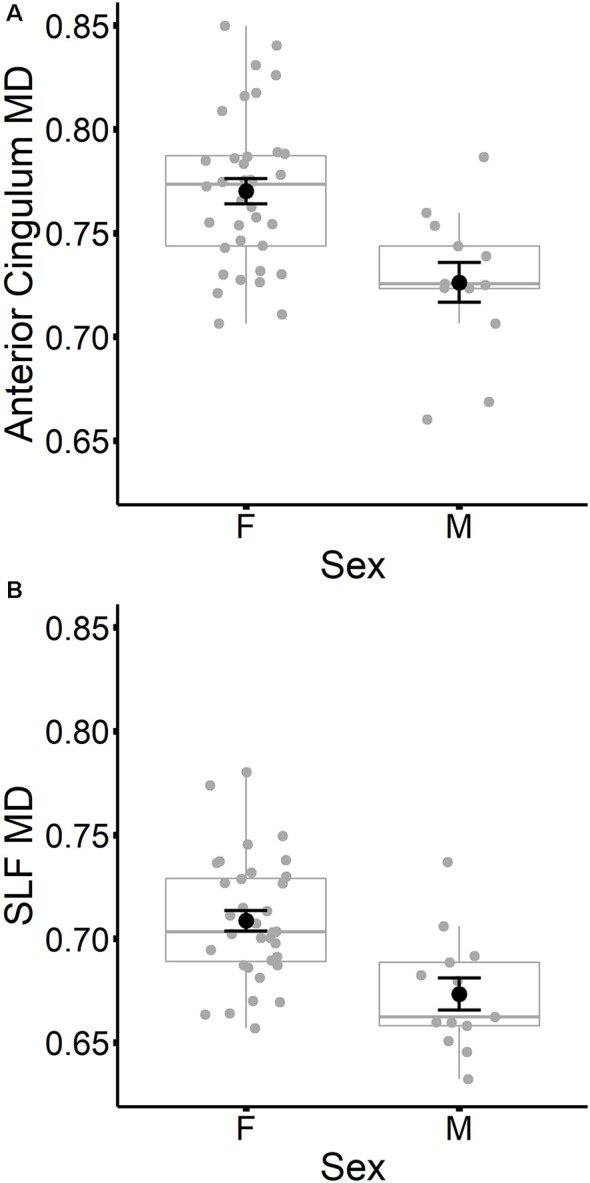
Mean diffusivity (MD) values at time 1 for the anterior cingulum **(A)** and SLF **(B)** are plotted for females and males separately. Individual participant values are represented as gray circles. Group means and error bars (plus/minus standard error) are shown in black.

In contrast to age, sex did not predict tract-specific FA values once global FA was accounted for, but remained a significant predictor of tract-specific MD values, even after controlling for global MD at time 1. To further explore the association between sex and MD in specific tracts, stepwise regressions were conducted for each tract. Global MD at time 1 was entered as a predictor into each regression model first, followed by sex. Results listed in Table 7 indicated that MD was higher for females compared to males in the anterior cingulum and SLF (depicted in Figure 3). After accounting for global MD, sex increased the variance explained by 16% and 15%, respectively, in these two regions. Although the stepwise regression also showed a significant effect of sex in the genu, we note that this effect did not remain significant after excluding a single outlier.

**Table 7:** Results for stepwise regressions performed on tract MD values (at time 1).

Tract	Predictors	β	*t*	*p*	*F*_(2,46)_	*Adj. R*^2^	*R*^2^ change
Genu*	Global MD	0.80	9.02	<0.001		
	Sex	−0.20	−2.24	0.03	41.25	0.63	0.04
Callosum body	Global MD	0.64	5.58	<0.001		
	Sex	−0.22	−1.93	n.s.	16.31	0.39	0.05
Splenium	Global MD	0.59	4.91	<0.001			
	Sex	−0.09	−0.74	n.s.	12.07	0.32	0.01
Fornix	Global MD	0.62	5.26	<0.001			
	Sex	−0.16	−1.33	n.s.	14.04	0.35	0.02
Anterior cingulum	Global MD	0.61	6.51	<0.001		
	**Sex**	**0.40**	**4.25**	<**0.001**	**34.49**	**0.58**	**0.16**
Posterior cingulum	Global MD	0.81	9.27	<0.001		
	Sex	−0.10	−1.17	n.s.	42.99	0.64	0.01
SLF	Global MD	0.69	8.54	<0.001			
	**Sex**	**0.39**	**4.79**	<**0.001**	**54.36**	**0.69**	**0.15**
IFOF	Global MD	0.69	6.42	<0.001			
	Sex	−0.09	−0.83	n.s.	20.59	0.45	0.01
Uncinate	Global MD	0.70	6.69	<0.001			
	Sex	0.05	0.47	n.s.	23.34	0.48	<0.01

*Global MD was entered into each model first, followed by age. Only results for the complete model (Global MD + Sex) are included here. Standardized β coefficients, *t*-statistics, and *p*-values are shown for Global MD and Sex predictors. The model-level *F* statistic and Adj. R^2^ are also reported. R^2^ change represents the amount of variance additionally explained by the model after adding Sex as a predictor. Statistically significant sex effects are bolded (except for the genu, as removing a single outlier value rendered this effect nonsignificant)*.

### Global FA and MD

The results of the MANOVAs suggested that the effects of most, but not all, of the independent predictors on tract-specific DTI measures were accounted for, once a global DTI metric was included as a covariate. We therefore conducted two additional mixed factor ANOVAs to better understand the relationship between global DTI metrics and the independent variables. These models included the same set of predictors as the tract-specific MANOVAs—age at time 1 (mean-centered), sex, APOE ε4 status, time, and the interactions between time and each between-subject variable. In the two separate ANOVAs, global FA and global MD at time 1 and time 2 were entered as the dependent variables.

**Figure 4:**
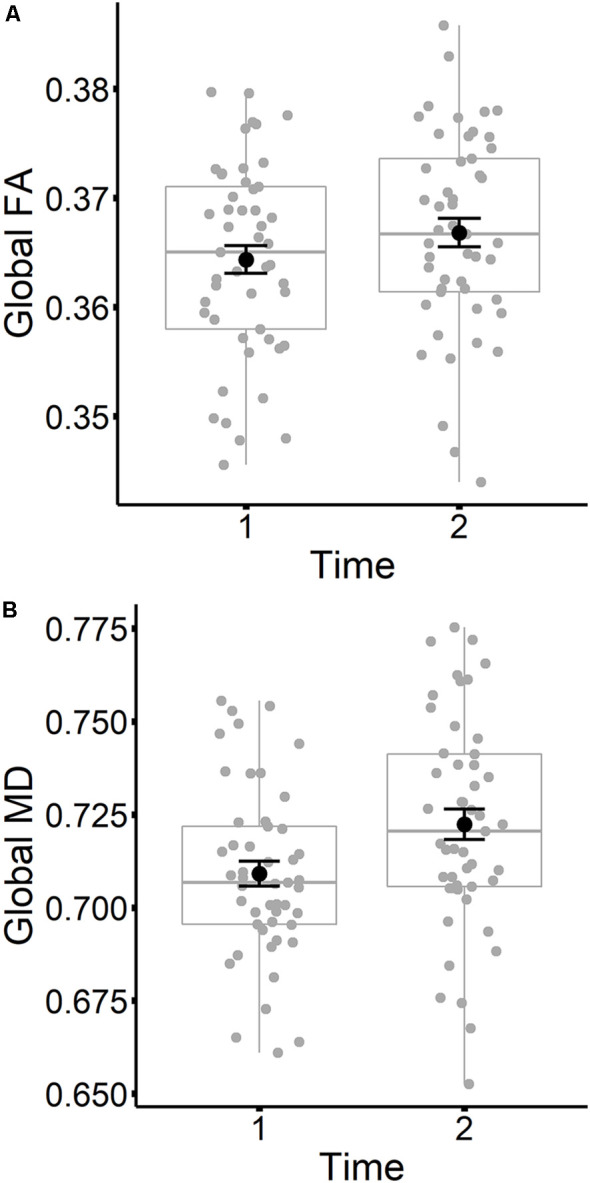
Global FA **(A)** and global MD **(B)** values are plotted for time 1 and time 2. Subject values are represented as gray circles. Group means and error bars (plus/minus standard error) are shown in black.

**Figure 5:**
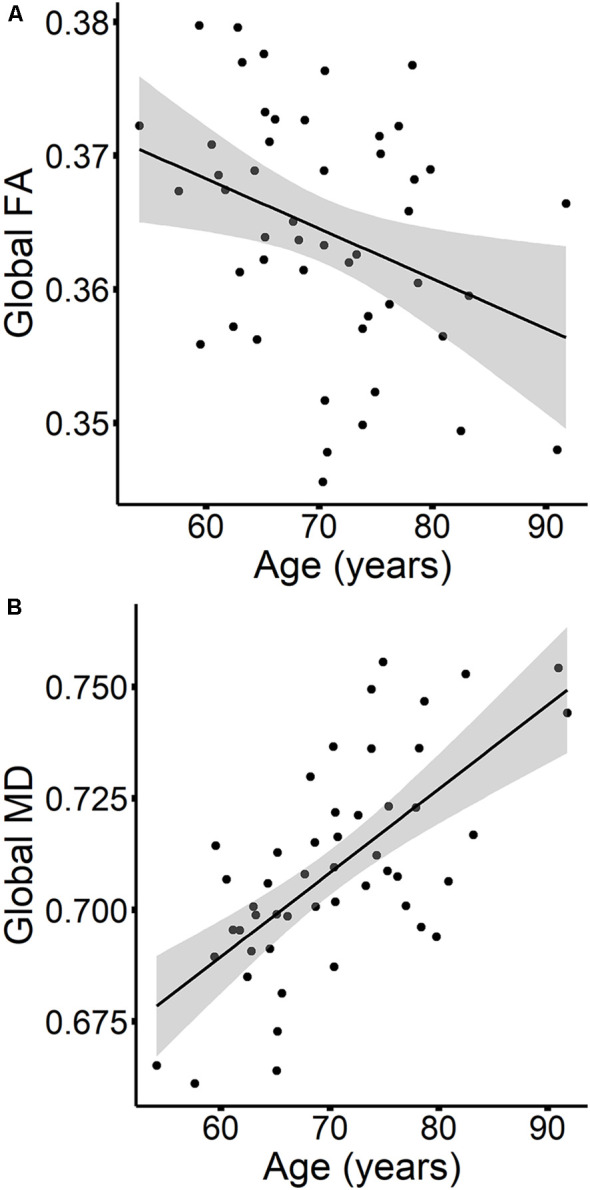
The relationships between age and global FA **(A)** and MD values **(B)** are presented in scatterplots, for time 1 values only. Confidence intervals are shown in gray.

**Figure 6:**
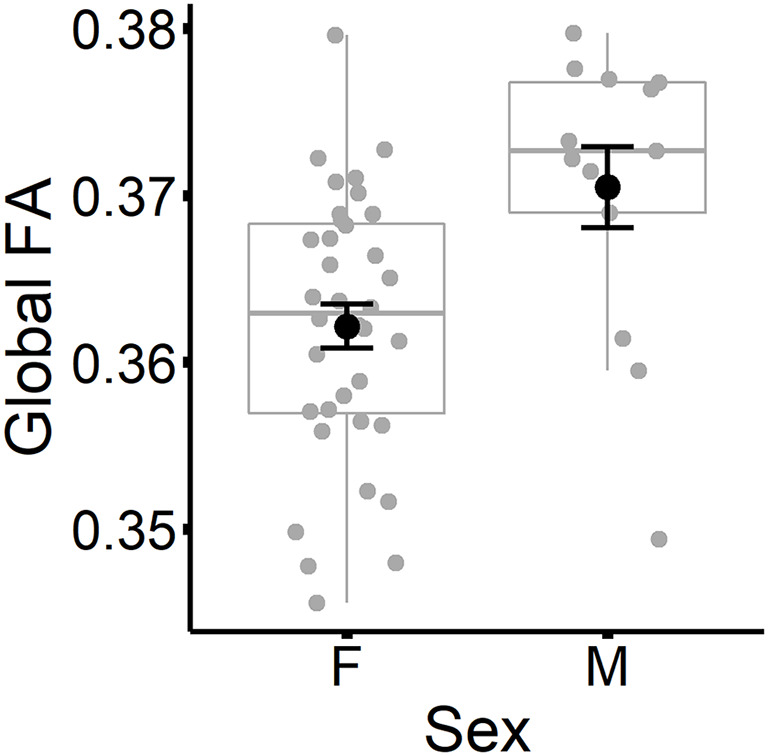
Global FA values (at time 1) are plotted for females and males separately. Subject values are represented as gray circles. Group means and error bars (plus/minus standard error) are shown in black.

The results are listed in Table 8. Results indicated a main effect of time on both global FA and global MD (see Figure 4). Follow-up paired *t*-tests revealed that global MD values were higher at the second time point (*t*_(48)_ = −4.79, *p* < 0.001). Unexpectedly, FA values were also higher at the second time point compared to time 1 (*t*_(48)_ = −3.33, *p* = 0.002). Age at time 1 also predicted global FA and global MD values (see Figure 5). Correlations between age at time 1 and global measures at time 1 showed lower global FA values (*r* = −0.35, *p* = 0.015) and higher global MD values (*r* = 0.67, *p* < 0.001) as age increased. Sex predicted global FA and showed a marginal effect on global MD (*p* = 0.051). Males showed higher global FA values at time 1 compared to females (*t*_(47)_ = 3.14, *p* = 0.003; see Figure 6). APOE ε4 status did not predict either global measure, and none of the interaction effects between time and other independent variables approached significance.

**Table 8:** Results of the mixed ANOVAs for global FA (left) and global MD (right) values.

	Global FA model			Global MD model		
Predictors	*F*	*p*	Pillai’s trace	*F*	*p*	Pillai’s trace
**Within-subject**
Time	4.84	0.033	0.10	15.64	<0.001	0.26
Time * Age	<0.001	n.s.	<0.01	0.70	n.s.	0.02
Time * Sex	1.92	n.s.	0.04	0.01	n.s.	<0.01
Time * APOE ε4	0.03	n.s.	<0.01	0.27	n.s.	0.01
**Between-subject**
Age	9.87	0.003	0.18	44.73	<0.001	0.50
Sex	11.02	0.002	0.20	4.03	n.s.	0.08
APOE ε4	0.07	n.s.	<0.01	0.35	n.s.	0.01

## Discussion

In this study, we investigated the influence of age, APOE ε4 status, and sex on white matter microstructure by examining changes in DTI metrics across two time-points separated by an average of 2.7 years. We sought to clarify the extent to which associations between our variables of interest and DTI metrics are best characterized as global or tract-specific. The results of this study provide evidence of a complex pattern of global and tract-specific white matter differences influenced by time, baseline age, and sex, but not APOE ε4 status, on FA and MD metrics. These relationships are discussed in detail in the following sections.

### APOE ε4 Did Not Predict FA or MD

We did not observe differences between APOE ε4 carriers and non-carriers in either tract-specific or global measures of FA and MD. This result is in contrast to evidence from cross-sectional (Persson et al., 2006; Smith et al., 2010; Heise et al., 2011; Lyall et al., 2014; Laukka et al., 2015; Cavedo et al., 2017) and longitudinal studies (Williams et al., 2019) linking APOE ε4 to reduced FA and increased MD values in white matter tracts, particularly those connecting frontal, temporal and parietal regions. However, other studies have failed to find an association with ε4 (Nyberg and Salami, 2014; Bendlin et al., 2010; Westlye et al., 2012; Ly et al., 2014; Rieckmann et al., 2016), suggesting that the relationship between APOE ε4 and DTI metrics is likely subtle. It is important to note that our participants were a subset of the cohort included in Ryan et al. (2011), who found cross-sectional differences in white matter FA and apparent diffusion coefficient (equivalent to MD) values between ε4 carriers and non-carriers in a larger sample of 126 participants, ages 52–92. The relationship to ε4 status was dependent on age, however, such that ε4 differences were more apparent at the older end of the age range. The notion that the effects of APOE ε4 become more obvious in late adulthood is consistent with the resource modulation hypothesis of genetic influences on aging (Lindenberger et al., 2008; Papenberg et al., 2015). By this view, age-associated losses in neurochemical or anatomical “brain resources” can strengthen genetic influences on cognition and brain structure/function. Thus, studies with larger sample sizes and wider age ranges may be necessary to detect the synergistic effects of age and ε4 on white matter microstructure. Larger sample sizes would also enable comparison of ε4 heterozygotes and homozygotes, and thus an exploration of potential dose-dependent effects of the APOE ε4 allele on white matter.

### Sex Predicted Global FA and Tract-Specific MD

We observed sex differences that were global for FA but suggested tract-specific differences for MD. Although our initial MANOVA suggested tract-specific associations between sex and FA, these were no longer significant after controlling for global FA. Males had higher global FA values compared to females. Other studies have shown higher FA values for males compared to females, both globally and within specific tracts (Lebel et al., 2010; Inano et al., 2011; van Hemmen et al., 2017; Ritchie et al., 2018). The present results suggest that sex differences in FA are best characterized as influencing white matter integrity across the whole brain rather than within specific tracts.

In contrast, the present results suggest that sex may have tract-specific associations to MD, in which values were lower for males compared to females in the anterior cingulum and SLF, even after controlling for global MD. Higher MD in the anterior cingulum and SLF for females compared to males has previously been observed within an older adult sample (Williams et al., 2019), as well as within additional tracts that included frontal-occipital tracts, the genu, the anterior portion of the corona radiata, and the posterior limb of the internal capsule. One interpretation of our tract-specific sex differences may be that males and females are subject to a different degree of partial volume effects, given that males generally have a larger intracranial brain volume. However, if this was the case, then we would expect to see the strongest sex differences in the thinner and smaller tracts, which are more susceptible to partial volume effects. Instead, we noted sex differences in the anterior cingulum and SLF, two of the largest tracts examined in this study. An alternative interpretation is that these two tracts are particularly sensitive to alterations in female hormones because of their connections to the anterior cingulate cortex (anterior cingulum) and prefrontal cortex (SLF). Anterior cingulate cortex volume has been shown to fluctuate throughout the menstrual cycle (De Bondt et al., 2013) and shows volumetric changes with the use of hormonal contraceptives (Pletzer et al., 2010; De Bondt et al., 2013) and hormone therapy (Zhang et al., 2016). Both animal and human studies suggest that estrogen regulates the activity of monoamines in the prefrontal cortex (Inagaki et al., 2010; Jacobs and D’Esposito, 2011) and that a decline in estrogen levels during menopause can have negative consequences for prefrontal cortical function (for review, see Shanmugan and Epperson, 2014). The vulnerabilities of these regions to alterations in female hormones could help account for sex differences in their associated white matter tracts. However, we did not observe sex differences in other tracts such as the fornix that also connect with hormone-sensitive limbic, hippocampal, and prefrontal areas (Catenaccio et al., 2016). Observing sex differences in smaller tracts like the fornix may require larger sample sizes than those included in the present study. While a single outlier value appeared to drive the effect of sex on the genu in our study, it is interesting to note that this result resembles observations from other studies of adult women having greater genu FA values compared to men (Oh et al., 2007; Kanaan et al., 2012, 2014).

Older women may be especially at risk for poorer white matter health due to metabolic disruptions that arise throughout menopause. Estrogen helps regulate glucose consumption to support the maintenance of bioenergetic systems in the brain (Rettberg et al., 2014). Therefore, when estrogen production declines during menopause, glucose metabolism in the brain is likewise impaired (Brinton et al., 2015). In this hypometabolic state, the brain may compensate by utilizing ketones as an energy resource (Riedel et al., 2016) which are converted from fatty acids—including the lipids that form white matter myelin. Supporting this hypothesis, Klosinski et al. (2015) observed white matter myelin catabolization occurring in aged female mice in response to menopause-induced glucose dysfunction, with axons showing signs of extensive myelin breakdown in the post-menopausal mice. The authors suggest that this phenomenon may be adaptive for maintaining neuronal function in the face of diminished glucose utilization, although perhaps only in the short-term. In humans, white matter catabolization may render older females more susceptible to neurological diseases that feature demyelination, such as Alzheimer’s Disease (Brinton et al., 2015). In the present study, we cannot say whether the sex differences we observed were due to changes associated with menopause, or reflect other differences in developmental trajectories, such as during adolescence. We view these findings as preliminary, in part because of the low number of males included within the sample, and acknowledge the need for replication. Clearly, this is an important and promising line of research that warrants further study, ideally through the use of longitudinal designs and the inclusion of broader lifespan samples.

### Age Predicted Global MD and Both Global and Tract-Specific FA

Age exerted robust global effects on MD and both global and tract-specific effects on FA. After controlling for global MD, the impact of baseline age on tract-specific MD values was non-significant, suggesting that the effects of age on MD were principally global, affecting white matter similarly throughout the cortex. Indeed, age alone accounted for 50% of the variance in global MD. The results for FA were more complicated. For global FA, baseline age also had a significant influence, although it accounted for a smaller portion of the variance (18%) compared to MD. Importantly, however, even after controlling for global FA, increasing age was associated with lower FA in multiple tracts including the genu and body of the corpus callosum, the anterior and posterior cingulum, and the IFOF. Age explained at minimum an additional 6% of the variance in FA values (IFOF), and up to 20% of the variance (posterior cingulum) beyond the variance explained by global FA. The results suggest that age influenced the integrity of white matter throughout the brain, but that certain tracts may be particularly vulnerable to the effects of age-related pathologies.

The global influence of age on MD and FA values may stem from the variety of cardiovascular, metabolic, and inflammatory processes that are commonly seen in late adulthood. In particular, cardiovascular risk factors (i.e., high blood pressure, high cholesterol, obesity) have been linked to DTI alterations in late adulthood (McEvoy et al., 2015; Fuhrmann et al., 2019; Williams et al., 2019), suggesting that poor cardiovascular health relates to impaired white matter integrity among middle-aged to older adults. There is also evidence linking white matter integrity to glucose metabolic dysfunction, as DTI alterations associated with type 2 diabetes diagnosis (Reijmer et al., 2013; Xiong et al., 2016; Sun et al., 2018; Zhuo et al., 2019) and measurably higher insulin resistance levels (Ryu et al., 2014) have been noted in studies of late adulthood. Beyond the global impact of age on white matter, the increased sensitivity of some tracts may be a consequence of variation in their structural composition. Regional differences in myelination levels have been proposed as one potential factor contributing to the differential vulnerability of white matter tracts to aging (Raz, 2000; Hoagey et al., 2019). For example, fibers in the genu are mostly thinner and less densely myelinated (Aboitiz et al., 1992; Björnholm et al., 2017) than fibers in the splenium. Multiple DTI studies have reported more robust associations between age and the genu compared to the splenium (Bendlin et al., 2010; Michielse et al., 2010; Bennett et al., 2010; Cox et al., 2016), consistent with the results of the present study.

Alternatively, it is important to consider that methodological issues may account for some of the tract-specific findings. ROI-based analyses are particularly sensitive to partial volume error, particularly in populations where atrophy is common, such as older adults. Differences in ROI size, tract size, or presence of crossing fibers can result in more or less sensitivity to partial volume error across tracts identified using *a priori* ROIs. These issues can affect the calculation of DTI metrics and artificially inflate variance among tracts. Utilizing a higher-resolution sequence, with a smaller voxel size and a greater number of diffusion gradients, would help to minimize partial volume effects in future studies. As well, replicating the present findings using alternative DTI methods including native-space fiber tracking or multivariate voxel-wise analyses that carry different advantages/disadvantages over ROI-based methods would help confirm a biological basis to these tract-specific age effects.

### Time Predicts Global Changes in MD and FA

Across the two timepoints sampled 2.7 years apart on average, we observed global changes in FA and MD. Once global DTI metrics were controlled for, there was no evidence of tract-specific changes over time. Consistent with prior research (de Groot et al., 2016; Storsve et al., 2016), we observed increases in global MD that mirrored our cross-sectional findings of a relationship between higher baseline age and elevated global MD. The lack of tract-specificity in MD changes, again consistent with our cross-sectional results, strengthens the argument that MD alterations due to aging occur in a widespread, regionally non-specific manner. Contrary to expectations and inconsistent with our cross-sectional associations with baseline age, global FA exhibited an increase over the time window. While surprising, Bender and Raz (2015) also observed similar FA increases over a two to three year period in the body of the corpus callosum across the adult lifespan and found that hypertension treatment duration moderated these changes. To our knowledge, no other study has reported age-associated increases in global FA longitudinally, and thus we take caution in interpreting this finding before replication.

The changes observed in MD and FA overtime did not vary across individuals concerning other factors included in the present study such as APOE ε4 status and sex, despite prior evidence that APOE ε4 status moderates age-related declines in DTI metrics (Ryan et al., 2011; Williams et al., 2019). Possibly, a period of 2–3 years is not sufficient to observe substantial alterations in white matter microstructural integrity associated with other moderating variables, particularly in a relatively healthy sample of older adults such as the present study. Alternatively, studies evaluating DTI metric changes over a similar length of time may require larger sample sizes, such as Bender and Raz (2015), who noted that trajectories over time were modified by health and genetic factors.

### Implications

In summary, we found that age exerts a strongly negative influence on white matter microstructural health that is widespread among association, limbic, and commissural tracts. However, there is considerable variation in the extent to which these tracts are affected by age, with some like the posterior cingulum more severely affected than others like the IFOF. We also observed poorer white matter health in females relative to males. The results of this study highlight the importance of considering global white matter when attempting to understand how predictive factors such as age, sex, or APOE ε4 status influence DTI metrics. Findings that initially appeared to be tract-specific, such as the effect of sex on FA, arose because of a strong association between the predictor and global white matter FA. Considering the impact of aging on FA, controlling for global FA was important in allowing us to more precisely characterize the sensitivity of individual tracts to age-related damage. Though not a standard practice, other DTI studies have similarly controlled for global DTI metrics to ensure the specificity of relationships between tract integrity and outcome measures by deriving the mean of DTI values across white matter (Bennett et al., 2015; Ngo et al., 2017; Memel et al., 2020), the mean of DTI values across the tracts of interest (Rabin et al., 2019), or a general factor of variance shared across tracts of interest (Penke et al., 2010; Cox et al., 2016). The results of the present study suggest that it may be important to account for global white matter DTI values when examining tract-specific associations between DTI metrics and variables such as age, sex, or APOE genotype. Future studies will be needed to replicate our results, ideally employing different methods and analytic approaches, to determine the strengths of each for determining the factors that influence global and tract-specific white matter variation.

## Data Availability Statement

The raw data supporting the conclusions of this article will be made available by the authors, without undue reservation.

## Ethics Statement

The studies involving human participants were reviewed and approved by University of Arizona’s Institutional Review Board. The patients/participants provided their written informed consent to participate in this study.

## Author Contributions

SM and LR designed the study, wrote, revised and approved the submitted manuscript. SM processed imaging data and performed the statistical analyses. All authors contributed to the article and approved the submitted version.

## Conflict of Interest

The authors declare that the research was conducted in the absence of any commercial or financial relationships that could be construed as a potential conflict of interest.

## Footnotes

^1^http://fsl.fmrib.ox.ac.uk

^2^http://dti-tk.sourceforge.net
